# Relevance of STIM/Orai Calcium Entry System Hyperactivation in Human Prostate Contractility in Benign Prostate Hyperplasia

**DOI:** 10.3390/ijms26188985

**Published:** 2025-09-15

**Authors:** José M. La Fuente, Mariam El Assar, Argentina Fernández, Leocadio Rodríguez-Mañas, Javier Angulo

**Affiliations:** 1 Serviço de Urologia, Centro Hospitalar e Universitário de Santo António (CHUdSA), 4099-001 Porto, Portugal; lafuentecarvalho@gmail.com; 2 Fundación para la Investigación Biomédica del Hospital de Getafe, 28905 Getafe, Spain; mariam.assar@salud.madrid.org; 3 Centro de Investigación Biomédica en Red de Fragilidad y Envejecimiento Saludable (CIBERFES), Instituto de Salud Carlos III, 28029 Madrid, Spain; leocadio.rodriguez@salud.madrid.org; 4 Instituto de Investigación IdiPaz, 28029 Madrid, Spain; 5 Servicio de Histología-Investigación, Unidad de Investigación Traslacional en Cardiología–IRYCIS/UFV, Hospital Universitario Ramón y Cajal, 28034 Madrid, Spain; argentina.fernandez@salud.madrid.org; 6 Servicio de Geriatría, Hospital Universitario de Getafe, 28905 Getafe, Spain

**Keywords:** benign prostate hyperplasia, lower urinary tract symptoms, human prostate, human bladder, store-operated calcium entry, stromal interaction molecule-1, Orai channels

## Abstract

Benign prostate hyperplasia (BPH) is characterized by prostate enlargement and dynamic alterations contributing to development of lower tract urinary symptoms (LUTS). Prostate hypercontractility has been proposed to contribute to BPH-related LUTS. The aim was to evaluate the effects of inhibiting stromal interaction molecule (STIM)/Orai calcium entry system on adrenergic and neurogenic contractions in prostate (HP) and bladder neck (HB) strips from BPH patients. Effects of STIM/Orai inhibition on adrenergic and neurogenic contractions of HP from organ donors (ODs) without BPH were also evaluated. HP and HB strips were obtained from 20 patients with BPH undergoing radical prostatectomy and from six OD at the time of organ collection for transplantation. Tissues were functionally evaluated for isometric tension recording. STIM-1, Orai1, and Orai3 protein expressions were determined in prostate tissues. STIM-1 was also localized by immunofluorescence in prostate sections. Norepinephrine-induced and neurogenic contractions were significantly reduced by STIM/Orai inhibition with YM-58483 (20 µM) in HP from BPH patients but not in tissues from ODs. STIM/Orai inhibition failed to significantly modify contraction of HB from BPH patients. Protein expression of STIM-1 was significantly elevated in HP from BPH patients. Functional contribution of STIM/Orai system to contractile tone is relevant in prostate when BPH is present, probably related to increased expression of STIM-1. Inhibition of STIM/Orai could have therapeutic implications for the management of BPH patients by alleviating prostatic hypercontraction.

## 1. Introduction

One of the most prevalent diseases in aging male population is benign prostatic hyperplasia (BPH) [1,2]. It is characterized by the proliferation of both stromal and glandular tissue that often results in prostate enlargement, especially involving the transition zone and periurethral glands [3]. BPH is a histological entity that progresses to a clinical condition when associated with lower urinary tract symptoms (LUTS). The prevalence of LUTS also increases with age in men. Around 20% of men suffer from moderate/severe LUTS in their fifth decade of life, increasing to 30% in their sixth and to 40% in the eighth decade [2].

Pathophysiology mechanisms of BPH leading to LUTS are not completely elucidated. Although prostate growth could obviously exert a mechanical impact on urethra and bladder neck complicating urine voiding function, severity of LUTS is not related to prostate size [4,5]. This fact highlights the relevance of dynamic processes contributing to symptomatology secondary to BPH driven by the contraction of smooth muscle of prostate stroma [6]. A positive correlation between urethral obstruction and prostatic smooth muscle content has been evidenced [7]. In fact, therapeutic approaches aimed at reducing prostatic smooth muscle contraction (α_1_-adrenergic antagonists) have been reported to be more effective than drugs inhibiting androgen-induced growth of the prostate (5-α-reductase inhibitors) in the reduction in urinary symptoms in BPH [6,8,9,10] and they now represent the first-line therapy for BPH symptomatology [11]. However, α_1_-adrenergic blockers do not display complete efficacy and are not free from adverse side effects [11,12]. This justifies the need to explore alternative therapeutic targets aiming to decrease prostatic smooth muscle contraction.

Calcium movements in smooth muscle cells are key events regulating contractile tone. Store-operated calcium entry (SOCE) process is critical in calcium homeostasis [13]. Stromal interaction molecule-1 (STIM-1)/Orai system importantly contributes to this process. STIM-1 proteins in the sarcoplasmic reticulum membrane oligomerize when intracellular calcium reservoirs are empty. Oligomerization facilitates the interaction of STIM-1 with plasmatic membrane triggering the activation of Orai selective calcium channels and allowing the entry of calcium into cytoplasm [14]. Alterations in STIM/Orai system functionality and expression have been recently associated with vascular complications and aging [15,16,17]. Notably, enhanced impacts of STIM/Orai inhibition on vascular contractility and/or relaxation have been related to vascular aging [15,16] and erectile dysfunction, especially in diabetes [17]. The functional upregulation of STIM/Orai in these situations has been linked to altered expression of the different elements of this calcium entry system, STIM-1, Orai1, and Orai3, with their relative contribution varying depending on the specific condition and vascular territory [15,16,17]. Among available modulators of SOCE, YM-58483, also known as BTP-2 (N-(4-(3,5-bis(trifluoromethyl)-1H-pyrazol-1-yl)phenyl)-4-methyl-1,2,3-thiadiazole-5-carboxamide) is a well-known blocker of STIM/Orai calcium entry system [18,19,20] that has been reported to be effective in vivo [21,22,23].

Although there is some evidence suggesting a potential involvement of STIM/Orai system in prostate cancer development and progression [24,25], to our knowledge, the role of this calcium entry system in the regulation of prostate smooth muscle contraction has not been elucidated. The aim of this study was to evaluate the functional effects of STIM/Orai inhibition on human prostate and bladder smooth muscle in BPH and to determine the impact of this condition on the protein expressions of STIM-1, Orai1, and Orai3 in prostate tissue.

## 2. Results

### 2.1. Study Subjects

Characteristics of study subjects from whom the tissues were collected are summarized in Table 1. Patients with BPH were significantly older and presented more cardiovascular risk factors although without reaching significant differences (Table 1). For BPH management, 18 (90%) patients were taking α_1_-blockers and 9 (45%) were taking 5-α-reductase inhibitors.

### 2.2. Inhibitor of STIM/Orai, YM-58483, Causes More Effective Relaxations in Prostate Strips from Patients with BPH

**Figure 1:**
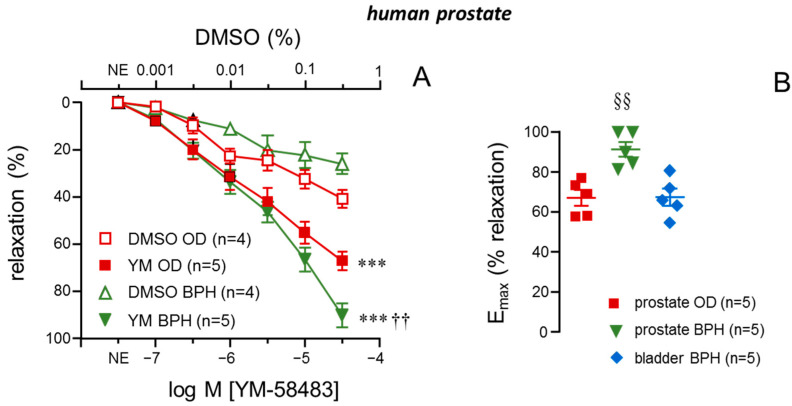
Inhibition of STIM/Orai calcium entry system causes enhanced relaxations of precontracted strips of human prostate in the presence of benign prostate hyperplasia (BPH). Panel (**A**) shows relaxations induced by cumulative additions of the vehicle (0.001% to 0.3% DMSO) or the STIM/Orai inhibitor, YM-58483 (YM, 0.1 to 30 µM) in human prostate strips from organ donors (ODs) or patients with BPH. Panel (**B**) shows maximum relaxation response induced by YM in human prostate strips from ODs and BPH patients as well as in human bladder neck strips from BPH patients. Data are displayed as mean ± S.E.M. of the percentage of relaxation induced by papaverine 0.1 mM at the end of the experiment. Individual data are also shown in panel B. *n* indicates the number of subjects from whom the tissues were obtained. *** *p* < 0.001 vs. respective vehicle, †† *p* < 0.01 vs. OD by a two-factors ANOVA test. §§ *p* < 0.01 vs. either prostate OD or bladder BPH by a one-factor ANOVA followed by Holm–Sidak’s test for multiple comparisons.

Increasing concentrations of YM-58483 (0.1 to 30 µM) caused relaxation of norepinephrine (NE)-precontracted prostate strips. This relaxant effect driven by STIM/Orai inhibition was significantly larger than that exerted by exposure to vehicle (0.001 to 0.3% DMSO) in strips from both OD subjects and BPH patients (Figure 1A). However, when YM-58483-induced responses were compared between tissues from organ donors and BPH patients, the relaxant effects of the STIM/Orai inhibitor were significantly more prominent in prostate strips from patients with BPH (Figure 1A). In fact, maximum relaxant responses to YM-58483 (E_max_) were significantly larger in prostate strips from patients with BPH (Figure 1B). Furthermore, evaluating relaxant responses to STIM/Orai inhibitor by determination of E_max_ revealed a significantly higher relaxant efficacy in prostate than in bladder strips, both obtained from BPH patients (Figure 1B).

### 2.3. STIM/Orai Inhibition Reduced NE-Induced Contractions in Prostate from BPH Patients

**Figure 2:**
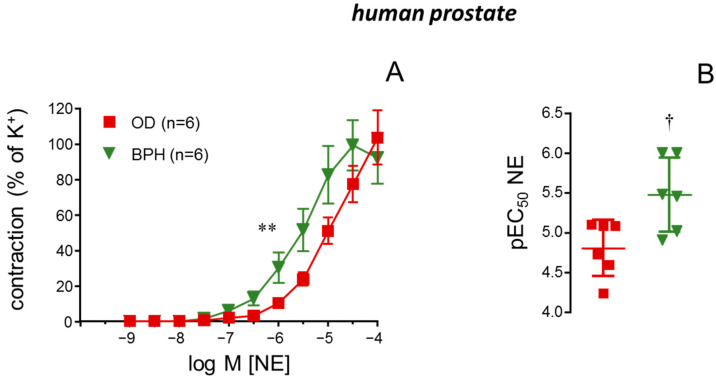
Sensitivity to adrenergic contraction in human prostate increases with benign prostate hyperplasia (BPH). Panel (**A**) shows contractions induced by cumulative additions of norepinephrine (NE, 1 nM to 100 µM) in human prostate strips from organ donors (ODs) or patients with BPH. Data are displayed as mean ± S.E.M. of the percentage of contraction caused by 125 mM K^+^. Panel (**B**) shows the values of pEC_50_ for NE in human prostate strips from ODs or patients with BPH. Data are shown as individual pEC_50_ values and the mean ± S.D. *n* designates the number of subjects from whom the tissues were obtained. ** *p* < 0.01 vs. OD by a two-factors ANOVA test, † *p* < 0.05 vs. OD by Mann–Whitney U test.

Cumulative additions of NE (1 nM to 100 µM) caused concentration-dependent contractions of human prostate strips from ODs and BPH patients. These contractions were significantly enhanced in strips from BPH patients (Figure 2A), which displayed increased sensitivity to NE as indicated by the significant augmentation of the pEC_50_ for NE in these tissues (4.81 ± 0.14, *n* = 6, vs. 5.48 ± 0.19, *n* = 6, for OD and BPH, respectively, *p* < 0.05) (Figure 2B). Considering that BPH patients were significantly older than OD subjects, we evaluated NE-induced contractions in OD subjects selected by being more than 50 years old (*n* = 4, average age 65.0 ± 6.7 years). Even considering only the prostate tissues from this specific population of older OD subjects, BPH tissues displayed a significant potentiation of the NE-induced contractions (Supplementary Figure S1A).

**Figure 3:**
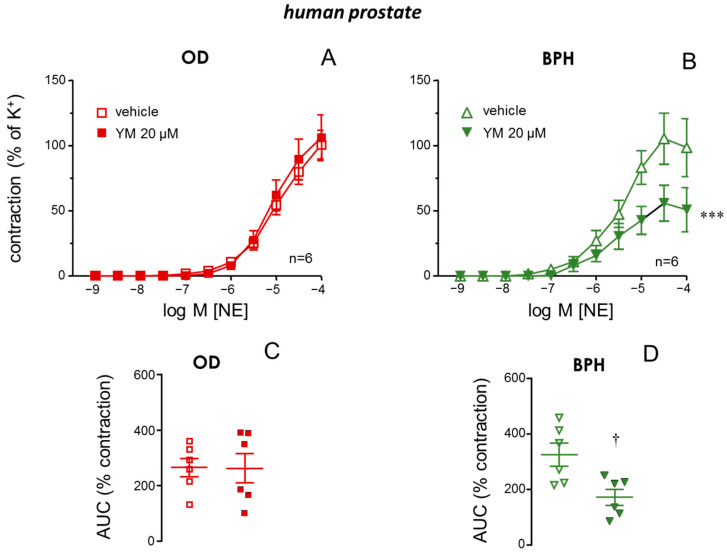
STIM/Orai inhibition reduces adrenergic contractions in prostate from patients with benign prostate hyperplasia (BPH). Superior panels show contractions induced by cumulative additions of norepinephrine (NE, 1 nM to 100 µM) in human prostate strips previously treated with vehicle (0.2% DMSO) or the STIM/Orai inhibitor, YM-58483 (YM, 20 µM) obtained from organ donors (ODs) (**A**) and from patients with BPH (**B**). Data are displayed as mean ± S.E.M. of the percentage of contraction caused by 125 mM K^+^. Lower panels show the values of the area under the curve (AUC) of NE-induced contractions in human prostate strips treated with vehicle or YM obtained from ODs (**C**) or patients with BPH (**D**). Data are shown as individual AUC values and the mean ± S.E.M. *n* designates the number of subjects from whom the tissues were obtained. *** *p* < 0.01 vs. vehicle by a two-factors ANOVA test, † *p* < 0.05 vs. vehicle by Mann–Whitney U test.

The STIM/Orai inhibitor, YM-58483 (20 µM) did not affect NE-induced contractions in tissues obtained from ODs (Figure 3A). In contrast, a significant reduction in NE-induced contractions by YM-58483 was observed in prostate tissues from patients with BPH (Figure 3B). Expressing contractile responses as the area under the curve (AUC) of contractions confirmed these observations since STIM/Orai inhibition resulted in significantly reduced AUC values in strips from BPH (Figure 3D) patients but not in those from ODs (Figure 3C). A similar lack of influence of STIM/Orai inhibition with YM-58483 was obtained when evaluating only prostate tissues from OD subjects older than 50 years old (Supplementary Figure S1B).

### 2.4. Neurogenic Contractions of the Prostate from BPH Patients Were Also Diminished by STIM/Orai Inhibition

**Figure 4:**
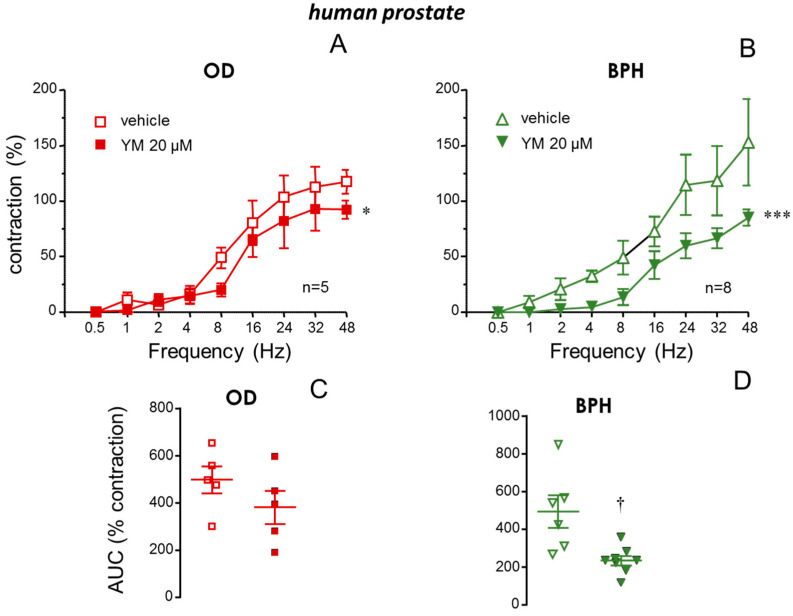
Neurogenic contractions of prostate from patients with benign prostate hyperplasia (BPH) are reduced by STIM/Orai inhibition. Superior panels show neurogenic contractions induced by electrical field stimulation (EFS, 0.5 to 48 Hz) in human prostate strips previously treated with vehicle (0.2% DMSO) or the STIM/Orai inhibitor, YM-58483 (YM, 20 µM) obtained from organ donors (ODs) (**A**) and from patients with BPH (**B**). Data are displayed as mean ± S.E.M. of the percentage of maximum contraction induced by EFS in control conditions. Lower panels show the values of the area under the curve (AUC) of contractions induced by EFS in human prostate strips treated with vehicle or YM obtained from ODs (**C**) or patients with BPH (**D**). Data are shown as individual AUC values and the mean ± S.E.M. *n* designates the number of subjects from whom the tissues were collected. * *p* < 0.05, *** *p* < 0.001 vs. vehicle by a two-factors ANOVA test, † *p* < 0.05 vs. vehicle by Mann–Whitney U test.

Neurogenic contractions of the prostate induced by electrical field stimulation (EFS) (0.5 to 48 Hz) were slightly but significantly reduced by the STIM/Orai inhibitor, YM-58483 (20 µM) in prostate tissues from ODs (Figure 4A). Nevertheless, a marked reduction in EFS-induced contractions was produced by YM-58483 in prostate strips obtained from BPH patients (Figure 4B). In fact, when EFS-induced contractions were expressed as AUC of contraction, significant reduction in AUC values was only observed when analyzing prostate tissues from patients with BPH (Figure 4C,D).

### 2.5. STIM/Orai Inhibition Did Not Modify Adrenergic nor Neurogenic Contractile Responses in Bladder Neck from BPH Patients

**Figure 5:**
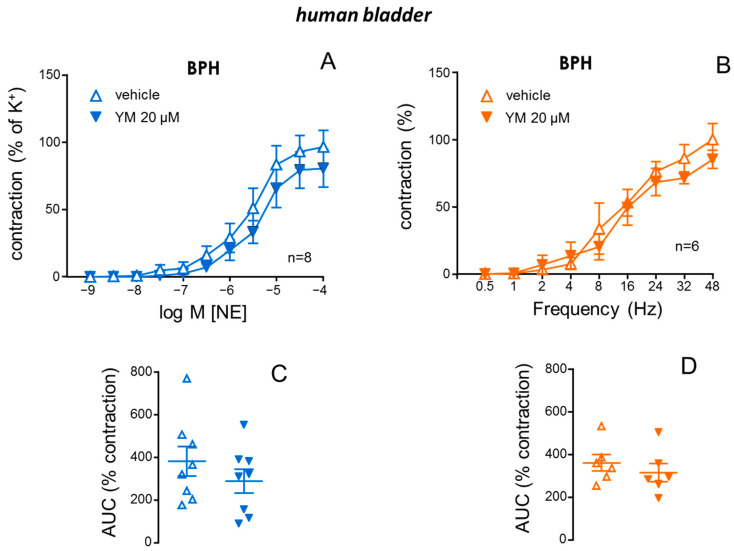
STIM/Orai inhibition failed to significantly modify adrenergic and neurogenic contractions in bladder neck from patients with benign prostate hyperplasia (BPH). Superior panels show contractions induced by cumulative additions of norepinephrine (NE, 1 nM to 100 µM) (**A**) and neurogenic contractions induced by electrical field stimulation (EFS, 0.5 to 48 Hz) (**B**) in human bladder neck strips previously treated with vehicle (0.2% DMSO) or the STIM/Orai inhibitor, YM-58483 (YM, 20 µM) obtained from patients with BPH. Data are displayed as mean ± S.E.M. of the percentage of contractions caused by 125 mM K^+^ (**A**) or the percentage of maximum contraction induced by EFS in control conditions (**B**). Lower panels show the values of the area under the curve (AUC) of NE- (**C**) and EFS-induced (**D**) contractions in human prostate strips treated with vehicle or YM obtained from patients with BPH. Data are shown as individual AUC values and the mean ± S.E.M. *n* indicates the number of subjects from whom the tissues were obtained.

In contrast to that observed in prostate tissue, NE-induced contractions were not significantly modified by the treatment with the STIM/Orai inhibitor, YM-58483 (20 µM), in strips of bladder neck obtained from BPH patients (Figure 5A). In the same sense, neurogenic contractions induced by EFS in bladder strips were not significantly affected by the STIM/Orai inhibitor, YM-58483 (Figure 5B). Moreover, the lack of impact of STIM/Orai inhibition on bladder contractility was confirmed by expressing the contractile responses as AUC. No significant alteration in these values by YM-58483 was evidenced with respect to adrenergic (Figure 5C) and neurogenic contractions (Figure 5D).

### 2.6. STIM-1 Was Upregulated in Prostate Tissue from Patients with BPH

**Figure 6:**
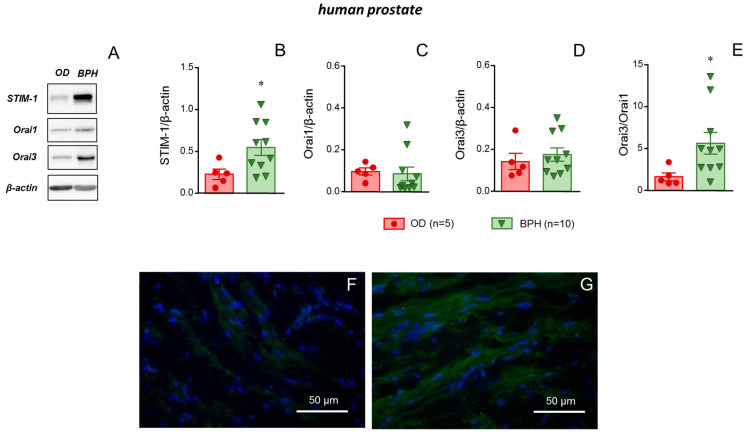
Benign prostate hyperplasia (BPH) is related to augmented expression of STIM-1 and increased Orai3/Orai1 ratio in prostate tissue. Panel (**A**) displays representative blots for STIM-1, Orai1, Orai3, and β-actin (loading control) proteins in human prostate tissue homogenates obtained from organ donors (ODs) and from patients with BPH. Other upper panels show quantification of the expression of STIM-1 (**B**), Orai1 (**C**), and Orai3 (**D**), as well as the ratio Orai3/Orai1 (**E**) in these tissues. Data are shown as individual protein/β-actin (**B**–**D**) or Orai3/Orai1 (**E**) values and the mean ± S.E.M. *n* indicates the number of subjects from whom the tissues were obtained. * *p* < 0.05 vs. ODs by Mann–Whitney U test. Representative images of immunofluorescence detection (green fluorescence) of STIM-1 in sections of human prostate from ODs and patients with BPH are shown in panels (**F**,**G**), respectively. Nuclei are counterstained in blue.

Immunodetection of the STIM/Orai system components, STIM-1, Orai1, and Orai3 in prostate homogenates revealed an upregulation of STIM-1 protein in samples from BPH patients with respect to those from ODs (Figure 6A). Quantification of immunoblots yielded a significant increase in STIM-1 protein expression in prostate tissue associated with the presence of BPH (Figure 6B) while protein contents of Orai1 and Orai3 were not significantly modified by BPH condition (Figure 6C,D). Interestingly, the relative content of the two Orai channels was altered since, despite the lack of individual modification, the ratio Orai3/Orai1 significantly increased in tissues from BPH patients (Figure 6E). There was no significant influence of BPH condition on β-actin expression in human prostatic tissues (Supplementary Figure S2). Protein expression of STIM-1 in prostate specimens was confirmed by immunofluorescence. STIM-1 was localized in smooth muscle cells of prostate tissue. The higher intensity observed in tissues from BPH patients appeared to be compatible with the upregulation of STIM-1 protein detected by Western blot (Figure 6F,G).

## 3. Discussion

The present results point to functional upregulation of STIM/Orai system playing a significant role in regulating prostatic smooth muscle tone in men with BPH. In contrast, the functional relevance of STIM/Orai system seems to be less significant in bladder contractility in these patients. This functional evidence is related to an increase in the sensor protein STIM-1 in the prostate tissue when BPH is present.

Although pathophysiological mechanisms leading to LUTS in patients with BPH are not completely elucidated, it is well established that, in addition to a mechanical effect driven by prostate growth, a dynamic component involving prostate smooth muscle contraction contributes to clinical symptoms [26]. In fact, a first-line treatment for BPH-related LUTS consists of pharmacological inhibition of α_1_-adrenergic receptors, which are demonstrated to reduce prostate smooth muscle contraction [27]. Thus, the availability of pharmacological alternatives modulating prostate contraction could help in managing patients with BPH-related LUTS. This concept is actually promoting research to find potential modulators of prostatic contractions [28,29,30] that could have significant clinical implications [31]. In this sense, the inhibition of calcium entry system, STIM/Orai, has been shown to modulate contractility and improve vascular function in different territories in the context of several conditions related to vascular impairment [15,16,17]. The present results reveal a role for STIM/Orai inhibition in modulating the contractility of human prostate in the presence of BPH. First, the addition of increasing concentrations of the STIM/Orai inhibitor, YM-58483, reversed the adrenergic-induced contraction in prostate strips from men with or without BPH but its significantly superior efficacy in prostate from BPH patients suggests an increased contribution of STIM/Orai in the contractile activity of smooth muscle prostate in BPH condition. In fact, this is also evidenced when comparing the maximum relaxant capacity of YM-58483. E_max_ for the STIM/Orai inhibitor is significantly higher in prostate from BPH patients compared to both prostate from ODs and bladder tissue from BPH patients. This suggests that, in the presence of BPH, the contribution of STIM/Orai to contraction is more relevant in the prostate than in the bladder. These concepts are reinforced by the ability of YM-58483 to reduce NE-induced contractions in prostate strips specifically obtained from patients with BPH, while it did not significantly influence adrenergic contractions in prostate tissues from healthy donors. This result was consistent with the marked inhibition of neurogenic contractions in prostate from BPH patients by the STIM/Orai inhibitor, which only exerted marginal effects on prostatic strips from ODs. Despite the scarce presence of α-adrenergic receptors in bladder detrusor smooth muscle, their functional relevance has been evidenced in the neck of human bladder [32]. Additionally, neurogenic contractions are sensitive to α-adrenergic receptor antagonism in human bladder neck [27]. Present results are consistent with this concept as suggested by the ability of NE to induce contractions of bladder neck strips from patients with BPH. Research evaluating the contribution of STIM/Orai system to bladder contractility is scarce, although the inhibition of this system has been proposed to reduce carbachol-induced contractions of human detrusor smooth muscle [33]. However, marked differences exist in the regulation of contractility in bladder detrusor with respect to bladder neck [34]. STIM/Orai inhibition failed to modify NE- or EFS-induced contractions in bladder neck from BPH patients, suggesting that the functional relevance of this calcium entry system in prostate contractility in BPH does not extend to bladder neck tissue.

Functional regulation of contractile tone of human prostate by STIM/Orai inhibition in BPH was associated with alteration in prostatic expression of elements of STIM/Orai system. Specifically, the sensor protein, STIM-1, was upregulated in prostate from BPH patients. Elevated expression of STIM-1 could potentially lead to hyperactivated STIM/Orai system, thereby explaining the functional effects of YM-58483 in prostate from BPH patients. This idea is supported by previous evidence showing that upregulation of STIM-1 expression was associated with functional hyperactivation of STIM/Orai system in rats with ethanol-induced hypertension [35]. Interestingly, in addition to STIM-1 upregulation, although the expressions of Orai1 and Orai3 are not significantly modified, the presence of BPH is related to a shift in their expression ratio, leading to a significant increase in the Orai3/Orai1 ratio. STIM-1 and Orai1 are the most extensively studied in both physiology and pathophysiology, but emerging evidence suggests a role for Orai3 channels in various pathophysiology conditions. Orai3 has been related to cancer development and poor prognosis [36,37], including prostate cancer [38], as well as in other conditions like vascular aging [16], vascular injury [39] and pulmonary fibrosis [40]. Thus, the increase in Orai3/Orai1 could be relevant to BPH pathophysiology. YM-58483-mediated inhibition of STIM/Orai system is considered a consequence of its blockade of Orai channels at extracellular level while STIM is in the endoplasmic reticulum [41]. However, YM-58483 does not show specificity for Orai subtypes [41]. The lack of using specific inhibitors together with the ability of Orai1 and Orai3 to generate heteromeric channels in prostatic tissue [24] precludes identification of the specific STIM/Orai element responsible for functional alterations in BPH prostate, which may be considered a limitation of this study. However, the evaluation of functional effects of STIM/Orai inhibitors and the expression of STIM/Orai elements in both healthy and pathological human prostate tissues should be considered a notable strength of the study.

Aging is the main risk factor for BPH, which is highly prevalent in older men [42]. The older age of BPH patients and the previously reported association of functional STIM/Orai hyperactivation with aging in other tissues [13,15,16] do not allow for establishing the specific factor, aging or BPH, responsible for STIM/Orai hyperactivation in prostate from BPH patients. This functional alteration in the calcium homeostasis system could be the result of BPH condition or aging itself. The fact that BPH-related alterations in adrenergic responses were still observed after selecting older OD subjects together with the lack of influence of STIM/Orai inhibition on such responses in this OD population point to a specific impact of BPH condition on STIM/Orai system in the prostate. However, the differences between the whole OD population and BPH patients with respect to age and comorbidities limit the strength of this assumption. In any case, most BPH patients are in older age and STIM/Orai inhibition could improve BPH-associated symptoms related to the dynamic component of the disease.

Finally, it has been demonstrated the key role of STIM and Orai in the calcium homeostasis of smooth muscle [20,43]. However, the lack of calcium measurements in human prostate tissue due to the limited availability of human specimens may also be considered as a limitation of the study.

## 4. Materials and Methods

### 4.1. Human Tissues

Specimens were obtained from patients undergoing suprapubic adenomectomy (Millin’s approach) for benign prostatic hyperplasia (BPH). One to three strips from peripheral prostate were dissected from each specimen for functional evaluation in organ chambers. A small specimen of bladder neck is also resected in the course of this type of surgery, which was used to dissect one to three bladder neck strips. For comparison, prostatic tissue specimens were collected from six organ donors at the moment of organ obtaining for transplantation. Patients or relatives gave their written informed consent before inclusion in the study. For transportation to the laboratory, the tissue specimens were immersed in M-400 solution (pH 7.4; 400 mOsm/kg. Composition in *w*/*v*: 4.19% mannitol, 0.2% KH_2_PO_4_, 0.97% K_2_HPO_4_·3 H_2_O, 0.11% KCl and 0.08% NaHCO_3_) and maintained at 0–4 °C until evaluation before 24 h [27,44]. The study followed the principles outlined in the Declaration of Helsinki and complied with European regulations regarding human tissue collection, conservation and elimination. Protocols and consent forms were approved by the Ethics Committee of Centro Hospitalar e Universitário de Santo António (CHUdSA), Porto, Portugal (2016.107(092-DEFI/089-CES) where the tissues were collected.

### 4.2. Functional Evaluation of Tissues in Organ Bath

Human prostate (HP) and bladder neck (HB) specimens were cleaned of fat and connective tissue and cut into strips for organ bath assays. HP and HB strips were placed in organ baths (8 mL) containing Krebs–Henseleit solution (KHS) and tied to force transducers. Composition of KHS was (in mM): NaCl 119, KCl 4.6, CaCl_2_ 1.5, MgSO_4_ 1.2, NaHCO_3_ 24.9, glucose 11, KH_2_PO_4_ 1.2, and EDTA 0.027. KHS was maintained at 37° C and perfused with 95% O_2_ and 5% CO_2_ to generate a pH of 7.4. Strips of prostate and bladder were maintained on 1.5 g resting tension for 90 min with extensive washouts for equilibration. After this period, strips were exposed to KKHS, a KHS containing 125 mM K^+^ by substituting NaCl with KCl KKHS-induced contraction was measured to check functionality and for normalization of contractile responses to NE. Relaxation efficacy of the STIM/Orai inhibitor, YM-58483 (Tocris, Bristol, UK) (0.1 to 30 µM), was evaluated in prostate and bladder neck strips precontracted with NE (Sigma-Aldrich, St. Louis, MO, USA) (1–10 µM). NE was dissolved at 10 mM concentration right before experiment in distilled water containing 0.1% ascorbic acid (Merck, Darmstadt, Germany) for preventing catechol oxidation. Serial dilutions were also made with distilled water containing 0.1% ascorbic acid. YM-58483 was dissolved in DMSO (Sigma-Aldrich) at 10 mM concentration. Further dilutions were made in distilled water.

EFS was produced by means of two platinum electrodes connected to an electrical stimulator (Cibertec, Madrid, Spain). EFS consisted of pulses of 75 mA of current intensity, 0.5 ms of pulse duration, in 20 s trains. EFS at increasing frequencies (0.5–48 Hz) was applied for evaluation of neurogenic contractions in human prostate and bladder neck strips under resting tension. Neurogenic nature of EFS-induced contractions with these parameters was previously evidenced [27,44].

### 4.3. Western Blot Experiments

Prostate tissues from ODs and patients with BPH were frozen in liquid nitrogen and stored at −80 °C until the extraction of proteins. Total protein content was obtained by homogenizing prostate tissues in T-PER lysis buffer (Pierce Biotechnology, Inc., Rockford, IL, USA) containing a Protease Inhibitor Cocktail (Roche Diagnostics, IN, USA) and following the instructions from manufacturer. The bicinchoninic acid (BCA) method was used for determination of protein content in prostate homogenates. SDS-PAGE on a 10% polyacrylamide gel was applied for separating protein extracts (20 µg). Transfer of proteins from gel to polyvinylidene difluoride membranes was then performed. Membranes were blocked for 5 min with EveryBlot blocking buffer (Bio-Rad, Hercules, CA, USA) and incubated at 4 °C overnight with specific mouse monoclonal antibodies against STIM-1 (cat.# MCA3475Z, Bio-Rad, 1:1000 dilution) or Orai1 (cat# MA5-15776, ThermoFisher Scientific, Waltham, MA, USA, 1:1000 dilution) or rabbit polyclonal antibody against Orai3 (cat# PA5-20370, ThermoFisher Scientific, 1:500 dilution). For loading control, a mouse antibody against β-actin (cat.# NB600-501, Novus Biologicals, Centennial, CO, USA, dilution 1:5000) was used. After washing with phosphate-buffered saline (PBS) containing 0.1% Tween, membranes were incubated for 60 min at room temperature with horseradish peroxidase-conjugated secondary antibodies: goat anti-rabbit (cat.# NB7160, Novus Biologicals, 1:10,000 dilution) for Orai3 or goat anti-mouse (cat.# NBP2-30347H, Novus Biologicals, 1:5000 dilution) for the other proteins. ECL detection system (ThermoFisher Scientific) was employed for visualization of blots. Band density quantification was accomplished by using QuantityOne/Chemi-Doc Image-Lab 6.0 Software (Bio-Rad). The ratio of band intensity for STIM-1, Orai1, or Orai3 relative to respective β-actin band intensity was utilized for the expression of data.

### 4.4. Immunofluorescence Assays

After immersing prostate tissues in increasing percentages of saccharose (10% to 30% *w*/*v*), the tissues were embedded in OCT and maintained at −80 °C until performing immunofluorescence assays [45]. Six μm thick sections of prostate tissue were obtained by cutting OCT blocks with a cryostat. Then, tissue sections were disposed on glass slides coated with polylysine. After removing the OCT, removal of autofluorescence and fixation of the tissues were accomplished by treating the sections with acetone and methanol. Then, sections were incubated at 4 °C overnight with mouse antibody against STIM-1 (cat.# MCA3475Z, Bio-Rad, 1:200 dilution). Sections were then washed with plus PBS containing 0.3% Triton X-100 and incubated with a secondary goat anti-mouse Alexa Fluor 488-conjugated secondary antibody (cat.# 1031-30, SouthernBiotech, Birmingham, AL, USA, 1:250 dilution). Nuclei were counterstained by incubating with diamidino-2-phenylindole (DAPI; Biorbyt, Cambridge, UK) for 60 min at room temperature. After mounting, sections were visualized in a fluorescence microscope (Olympus BX51, Olympus Corporation, Tokyo, Japan).

### 4.5. Data Analysis

Data are expressed as mean ± S.E.M. of the percentage of K^+^-induced contraction for concentration-dependent curves and of the percentage of maximum contraction previously induced by EFS in control conditions for frequency–response curves. Two-factors analysis of variance (ANOVA) was used to compare complete curves. pEC_50_ is defined as the −log M of the concentration required for obtaining 50% of K^+^-induced contraction. E_max_ is defined as the maximum response. Sum of percentages of contraction along the concentration–response curve was determined for calculating the area under the curve (AUC) of the contraction responses. Mann–Whitney U test or one-factor ANOVA followed by a Holm–Sidak’s test (multiple comparisons) were employed for analyzing these data. Descriptive data of BPH patients and OD subjects were compared by Fisher’s exact test for categorical variables and by Mann–Whitney U test for age. Each graph displays the number of subjects used for specific determinations. A probability value < 0.05 was considered significant in all cases. GraphPad Prism software (6.0 version, San Diego, CA, USA) was used for the analyses.

## 5. Conclusions

Pharmacological inhibition of STIM/Orai calcium entry system modulates adrenergic and neurogenic contractions of human prostate smooth muscle when BPH is present. This effect is related to upregulation of STIM-1 and increased Orai3/Orai1 ratio in BPH prostates, suggesting that altered expression of STIM/Orai elements promotes hyperactivation of this system in BPH. Notably, this functional hyperactivation of STIM/Orai by BPH does not appear to occur in the bladder neck. The relevance of the STIM/Orai system in regulating prostate smooth muscle tone in BPH could have potential therapeutic implications for managing these patients and it represents a kickoff for future research.

## Figures and Tables

**Table 1:** Characteristics of study subjects.

	OD	BPH	*p* Value
*n*	6	20	
Age (years)	54.5 ± 18.7	70.2 ± 5.5	**0.0436**
Diabetes (%)	0 (0.0)	5 (25.0)	0.2981
Dyslipidemia (%)	0 (0.0)	4 (20.0)	0.5425
Hypertension (%)	1 (12.5)	8 (40.0)	0.3798
Obesity (%)	0 (0.0)	0 (0.0)	1.0000
Smoking habit (%)	0 (0.0)	4 (20.0)	0.3214

BPH: patients with benign prostate hyperplasia; OD: organ donors without notice of BPH. *n* indicates number of subjects. Age is expressed as mean ± S.D. and was compared by Mann–Whitney U test. Fisher’s exact test was used to compare other variables between two groups. Significant differences are highlighted in bold.

## Data Availability

The data presented in this study are available on request from the corresponding author. The data are not publicly available due to data protection policy.

## Supplementary Materials

The following supporting information can be downloaded at https://www.mdpi.com/article/10.3390/ijms26188985/s1, Figure S1: Similar results on adrenergic responses of human prostate remained when considering only older organ donors. Figure S2: Benign prostate hyperplasia (BPH) did not significantly modify β-actin expression in human prostate. Figure S3: Complete images of immunoblots for STIM-1, Orai1, Orai3 and β-actin in human prostate.

## Abbreviations

The following abbreviations are used in this manuscript:

AUC	Area under the curve
BPH	Benign prostate hyperplasia
DMSO	Dimethyl sulfoxide
Emax	Maximum response
HB	Human bladder
HP	Human prostate
LUTS	Lower urinary tract symptoms
NE	Norepinephrine
OD	Organ donor
PBS	Phosphate-buffered saline
pEC50	−log M of the concentration required for 50% of contraction
SOCE	Store-operated calcium entry
STIM-1	Stromal interaction protein-1
YM	YM-58483

## References

[1] Soler R., Andersson K.E., Chancellor M.B., Chapple C.R., de Groat W.C., Drake M.J., Gratzke C., Lee R., Cruz F. (2013). Future direction in pharmacotherapy for non-neurogenic male lower urinary tract symptoms. Eur. Urol..

[2] Madersbacher S., Sampson N., Culig Z. (2019). Pathophysiology of Benign Prostatic Hyperplasia and Benign Prostatic Enlargement: A Mini-Review. Gerontology.

[3] Roehrborn C.G. (2005). Benign prostatic hyperplasia: An overview. Rev. Urol..

[4] Eckhardt M.D., van Venrooij G.E., Boon T.A. (2001). Symptoms and quality of life versus age, prostate volume, and urodynamic parameters in 565 strictly selected men with lower urinary tract symptoms suggestive of benign prostatic hyperplasia. Urology.

[5] Eckhardt M.D., van Venrooij G.E., Boon T.A. (2001). Symptoms, prostate volume, and urodynamic findings in elderly male volunteers without and with LUTS and in patients with LUTS suggestive of benign prostatic hyperplasia. Urology.

[6] Lepor H. (2007). Alpha blockers for the treatment of benign prostatic hyperplasia. Rev. Urol..

[7] Shapiro E., Hartanto V., Lepor H. (1992). The response to alpha blockade in benign prostatic hyperplasia is related to the percent area density of prostate smooth muscle. Prostate.

[8] Rigatti P., Brausi M., Scarpa R.M., Porru D., Schumacher H., Rizzi C.A., MICTUS Study Group (2003). A comparison of the efficacy and tolerability of tamsulosin and finasteride in patients with lower urinary tract symptoms suggestive of benign prostatic hyperplasia. Prostate Cancer Prostatic Dis..

[9] Hasan M., Parveen F., Shamsuzzaman A.K., Kibria M.D. (2007). Comparison of efficacy between Tamsulosin and Finasteride on symptomatic Benign Prostatic Hyperplasia. Mymensingh Med. J..

[10] Ventura S., Oliver V.I., White C.W., Xie J.H., Haynes J.M., Exintaris B. (2011). Novel drug targets for the pharmacotherapy of benign prostatic hyperplasia (BPH). Br. J. Pharmacol..

[11] Yu Z.J., Yan H.L., Xu F.H., Chao H.C., Deng L.H., Xu X.D., Huang J.B., Zeng T. (2020). Efficacy and Side Effects of Drugs Commonly Used for the Treatment of Lower Urinary Tract Symptoms Associated with Benign Prostatic Hyperplasia. Front. Pharmacol..

[12] Müderrisoglu A.E., de la Rosette J.J.M.C.H., Michel M.C. (2023). Potential side effects of currently available pharmacotherapies in male lower urinary tract symptoms suggestive of benign prostatic hyperplasia. Expert. Opin. Drug Saf..

[13] Collins H.E., Zhang D., Chatham J.C. (2022). STIM and Orai Mediated Regulation of Calcium Signaling in Age-Related Diseases. Front. Aging.

[14] Kodakandla G., Akimzhanov A.M., Boehning D. (2023). Regulatory mechanisms controlling store-operated calcium entry. Front. Physiol..

[15] El Assar M., García-Rojo E., Sevilleja-Ortiz A., Sánchez-Ferrer A., Fernández A., García-Gómez B., Romero-Otero J., Rodríguez-Mañas L., Angulo J. (2022). Functional Role of STIM-1 and Orai1 in Human Microvascular Aging. Cells.

[16] Sevilleja-Ortiz A., El Assar M., García-Rojo E., García-Gómez B., Fernández A., Sánchez-Ferrer A., La Fuente J.M., Romero-Otero J., Rodríguez-Mañas L., Angulo J. (2021). Ageing-induced hypercontractility is related to functional enhancement of STIM/Orai and upregulation of Orai 3 in rat and human penile tissue. Mech. Ageing Dev..

[17] Sevilleja-Ortiz A., El Assar M., García-Gómez B., La Fuente J.M., Alonso-Isa M., Romero-Otero J., Martínez-Salamanca J.I., Fernández A., Rodríguez-Mañas L., Angulo J. (2022). STIM/Orai Inhibition as a Strategy for Alleviating Diabetic Erectile Dysfunction Through Modulation of Rat and Human Penile Tissue Contractility and in vivo Potentiation of Erectile Responses. J. Sex. Med..

[18] Ishikawa J., Ohga K., Yoshino T., Takezawa R., Ichikawa A., Kubota H., Yamada T. (2003). A pyrazole derivative, YM-58483, potently inhibits store-operated sustained Ca2+ influx and IL-2 production in T lymphocytes. J. Immunol..

[19] Ohga K., Takezawa R., Yoshino T., Yamada T., Shimizu Y., Ishikawa J. (2008). The suppressive effects of YM-58483/BTP-2, a store-operated Ca2+ entry blocker, on inflammatory mediator release in vitro and airway responses in vivo. Pulm. Pharmacol. Ther..

[20] Masson B., Le Ribeuz H., Sabourin J., Laubry L., Woodhouse E., Foster R., Ruchon Y., Dutheil M., Boët A., Ghigna M.R. (2022). Orai1 Inhibitors as Potential Treatments for Pulmonary Arterial Hypertension. Circ. Res..

[21] Zhang W., Qi Z., Wang Y. (2017). BTP2, a Store-Operated Calcium Channel Inhibitor, Attenuates Lung Ischemia-Reperfusion Injury in Rats. Inflammation.

[22] Miyoshi M., Liu S., Morizane A., Takemasa E., Suzuki Y., Kiyoi T., Maeyama K., Mogi M. (2018). Efficacy of constant long-term delivery of YM-58483 for the treatment of rheumatoid arthritis. Eur. J. Pharmacol..

[23] Nehme A., Ghahramanpouri M., Ahmed I., Golsorkhi M., Thomas N., Munoz K., Abdipour A., Tang X., Wilson S.M., Wasnik S. (2022). Combination therapy of insulin-like growth factor I and BTP-2 markedly improves lipopolysaccharide-induced liver injury in mice. FASEB J..

[24] Ardura J.A., Álvarez-Carrión L., Gutiérrez-Rojas I., Alonso V. (2020). Role of Calcium Signaling in Prostate Cancer Progression: Effects on Cancer Hallmarks and Bone Metastatic Mechanisms. Cancers.

[25] Daba M.Y., Fan Z., Li Q., Yuan X., Liu B. (2023). The role of calcium channels in prostate cancer progression and potential as a druggable target for prostate cancer treatment. Crit. Rev. Oncol. Hematol..

[26] Shan S., Su M. (2025). The role of RhoA-ROCK signaling in benign prostatic hyperplasia: A review. Hum. Cell.

[27] Angulo J., Cuevas P., Fernández A., La Fuente J.M., Allona A., Moncada I., Sáenz de Tejada I. (2012). Tadalafil enhances the inhibitory effects of tamsulosin on neurogenic contractions of human prostate and bladder neck. J. Sex. Med..

[28] Huang R., Liu Y., Hu S., Tamalunas A., Waidelich R., Strittmatter F., Stief C.G., Hennenberg M. (2022). Inhibition of α1-Adrenergic, Non-Adrenergic and Neurogenic Human Prostate Smooth Muscle Contraction and of Stromal Cell Growth by the Isoflavones Genistein and Daidzein. Nutrients.

[29] Wang R., Huang R., Liu Y., Tamalunas A., Stief C.G., Hennenberg M. (2023). Silencing of CDC42 inhibits contraction and growth-related functions in prostate stromal cells, which is mimicked by ML141. Life Sci..

[30] Bester B., Koslowa K., Gronau A.C., Mietens A., Nowell C., Whittaker M.R., Pilatz A., Wagenlehner F., Exintaris B., Middendorff R. (2024). The oxytocin antagonist cligosiban reduces human prostate contractility: Implications for the treatment of benign prostatic hyperplasia. Br. J. Pharmacol..

[31] Hu S., Müderrisoglu A.E., Ciotkowska A., Kale O., Keller P., Schott M., Tamalunas A., Waidelich R., Stief C.G., Hennenberg M. (2024). Effects of carvedilol on human prostate tissue contractility and stromal cell growth pointing to potential clinical implications. Pharmacol. Rep..

[32] Michel M.C., Vrydag W. (2006). Alpha1-, alpha2- and beta-adrenoceptors in the urinary bladder, urethra and prostate. Br. J. Pharmacol..

[33] Luptak J., Kocmalova M., Franova S., Sutovsky J., Grendar M., Svihra J., Kliment J., Dusenka R., Sutovska M. (2018). Involvement of calcium regulating ion channels in contractility of human isolated urinary bladder. Gen. Physiol. Biophys..

[34] Michel M.C., Barendrecht M.M. (2008). Physiological and pathological regulation of the autonomic control of urinary bladder contractility. Pharmacol. Ther..

[35] Souza Bomfim G.H., Mendez-Lopez I., Arranz-Tagarro J.A., Ferraz Carbonel A.A., Roman-Campos D., Padín J.F., Garcia A.G., Jurkiewicz A., Jurkiewicz N.H. (2017). Functional Upregulation of STIM-1/Orai-1-Mediated Store-Operated Ca2+ Contributing to the Hypertension Development Elicited by Chronic EtOH Consumption. Curr. Vasc. Pharmacol..

[36] Kim J.H., Hwang K.H., Oh J., Kim S.E., Lee M.Y., Lee T.S., Cha S.K. (2025). Differential expression of ORAI channels and STIM proteins in renal cell carcinoma subtypes: Implications for metastasis and therapeutic targeting. Korean J. Physiol. Pharmacol..

[37] Nguyen A., Sung Y., Lee S.H., Martin C.E., Srikanth S., Chen W., Kang M.K., Kim R.H., Park N.H., Gwack Y. (2023). Orai3 Calcium Channel Contributes to Oral/Oropharyngeal Cancer Stemness through the Elevation of ID1 Expression. Cells.

[38] Kouba S., Buscaglia P., Guéguinou M., Ibrahim S., Félix R., Guibon R., Fromont G., Pigat N., Capiod T., Vandier C. (2023). Pivotal role of the ORAI3-STIM2 complex in the control of mitotic death and prostate cancer cell cycle progression. Cell. Calcium.

[39] Wu Q., Fang Y., Huang X., Zheng F., Ma S., Zhang X., Han T., Gao H., Shen B. (2023). Role of Orai3-Mediated Store-Operated Calcium Entry in Radiation-Induced Brain Microvascular Endothelial Cell Injury. Int. J. Mol. Sci..

[40] Yu C., Zhou Z., Huang W., Li X., Zou F., Meng X., Cai S. (2022). Orai3 mediates Orai channel remodelling to activate fibroblast in pulmonary fibrosis. J. Cell. Mol. Med..

[41] Zhang X., Xin P., Yoast R.E., Emrich S.M., Johnson M.T., Pathak T., Benson J.C., Azimi I., Gill D.L., Monteith G.R. (2020). Distinct pharmacological profiles of ORAI1, ORAI2, and ORAI3 channels. Cell. Calcium.

[42] Huang Q., Li B.H., Wang Y.B., Zi H., Zhang Y.Y., Li F., Fang C., Tang S.D., Jin Y.H., Huang J. (2024). Clinical biomarker-based biological aging and risk of benign prostatic hyperplasia: A large prospective cohort study. Aging Med. (Milton).

[43] Mancarella S., Potireddy S., Wang Y., Gao H., Gandhirajan R.K., Autieri M., Scalia R., Cheng Z., Wang H., Madesh M. (2013). Targeted STIM deletion impairs calcium homeostasis, NFAT activation, and growth of smooth muscle. FASEB J..

[44] La Fuente J.M., Fernández A., Cuevas P., González-Corrochano R., Chen M.X., Angulo J. (2014). Stimulation of large-conductance calcium-activated potassium channels inhibits neurogenic contraction of human bladder from patients with urinary symptoms and reverses acetic acid-induced bladder hyperactivity in rats. Eur. J. Pharmacol..

[45] Angulo J., El Assar M., Sevilleja-Ortiz A., Fernández A., Sánchez-Ferrer A., Romero-Otero J., Martínez-Salamanca J.I., La Fuente J.M., Rodríguez-Mañas L. (2019). Short-term pharmacological activation of Nrf2 ameliorates vascular dysfunction in aged rats and in pathological human vasculature. A potential target for therapeutic intervention. Redox Biol..

