# Supplementary material for: Relevance of STIM/Orai Calcium Entry System Hyperactivation in Human Prostate Contractility in Benign Prostate Hyperplasia
Source: Int J Mol Sci. 2025 Sep 15;26(18):8985. doi: 10.3390/ijms26188985 (PMC12469369; doi:10.3390/ijms26188985)
Supplement: Supplementary file 1 [file ijms-26-08985-s001.zip › ijms-3831346-supplementary.pptx]

## Slide 1
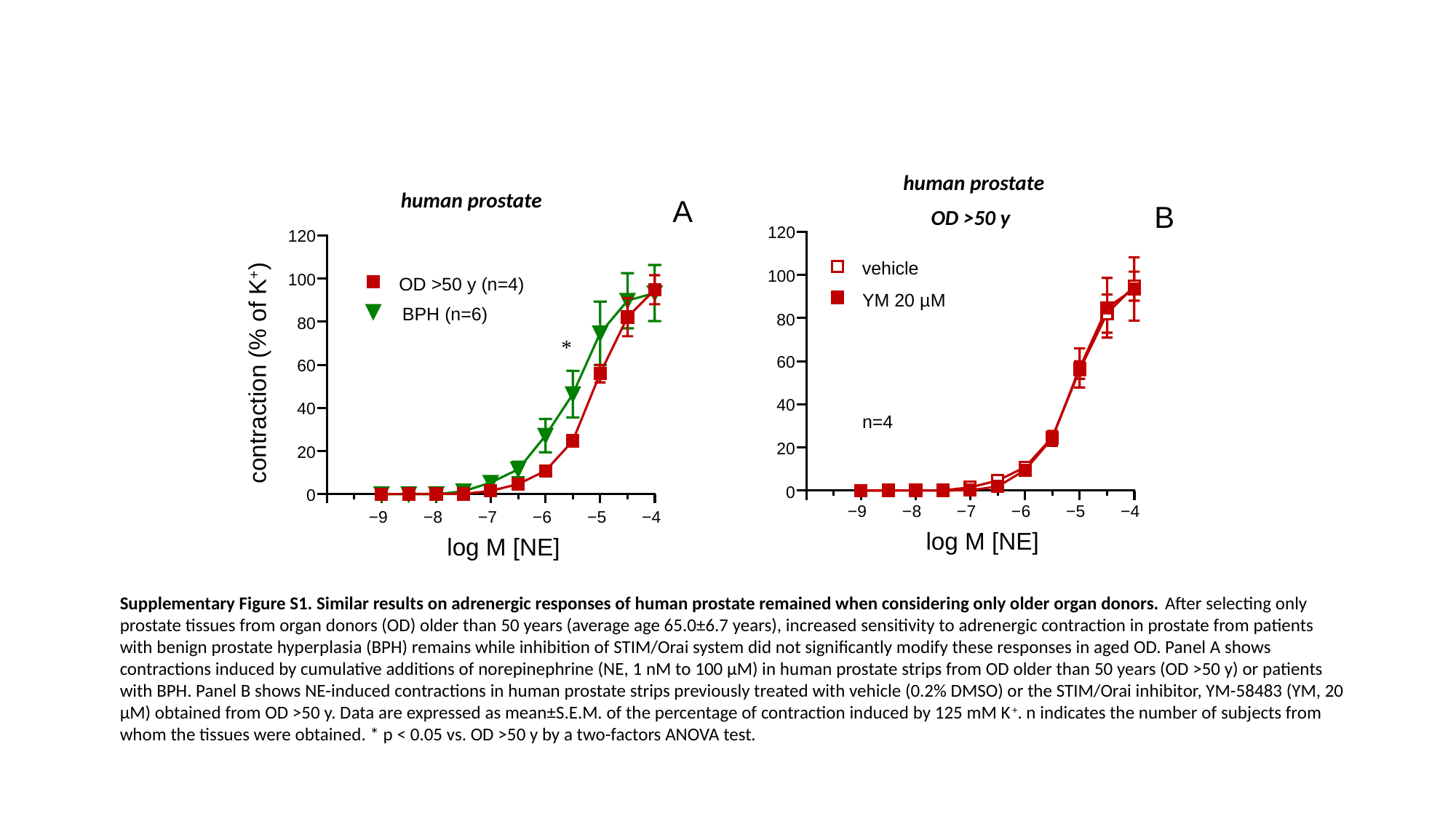

human prostate
A
human prostate
B
OD >50 y
120
120
vehicle
100
100
OD >50 y (n=4)
YM 20 µM
BPH (n=6)
80
80
*
60
60
contraction (% of K+)
40
40
n=4
20
20
0
0
−9
−8
−7
−6
−5
−4
log M [NE]
−9
−8
−7
−6
−5
−4
log M [NE]
Supplementary Figure S1. Similar results on adrenergic responses of human prostate remained when considering only older organ donors. After selecting only prostate tissues from organ donors (OD) older than 50 years (average age 65.0±6.7 years), increased sensitivity to adrenergic contraction in prostate from patients with benign prostate hyperplasia (BPH) remains while inhibition of STIM/Orai system did not significantly modify these responses in aged OD. Panel A shows contractions induced by cumulative additions of norepinephrine (NE, 1 nM to 100 µM) in human prostate strips from OD older than 50 years (OD >50 y) or patients with BPH. Panel B shows NE-induced contractions in human prostate strips previously treated with vehicle (0.2% DMSO) or the STIM/Orai inhibitor, YM-58483 (YM, 20 µM) obtained from OD >50 y. Data are expressed as mean±S.E.M. of the percentage of contraction induced by 125 mM K+. n indicates the number of subjects from whom the tissues were obtained. * p < 0.05 vs. OD >50 y by a two-factors ANOVA test.

## Slide 2
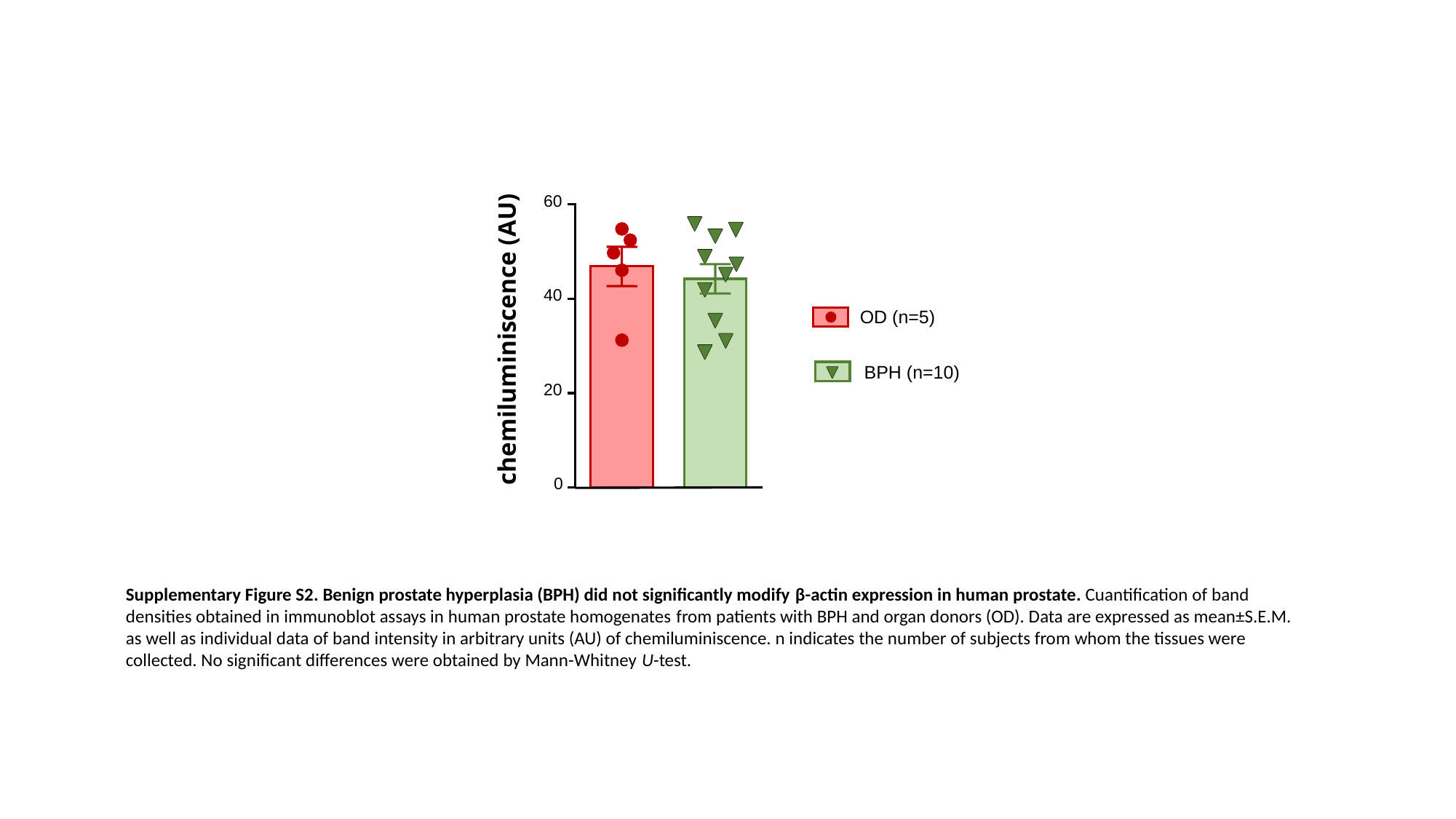

60
40
20
0
OD (n=5)
chemiluminiscence (AU)
BPH (n=10)
Supplementary Figure S2. Benign prostate hyperplasia (BPH) did not significantly modify β-actin expression in human prostate. Cuantification of band densities obtained in immunoblot assays in human prostate homogenates from patients with BPH and organ donors (OD). Data are expressed as mean±S.E.M. as well as individual data of band intensity in arbitrary units (AU) of chemiluminiscence. n indicates the number of subjects from whom the tissues were collected. No significant differences were obtained by Mann-Whitney U-test.

## Slide 3
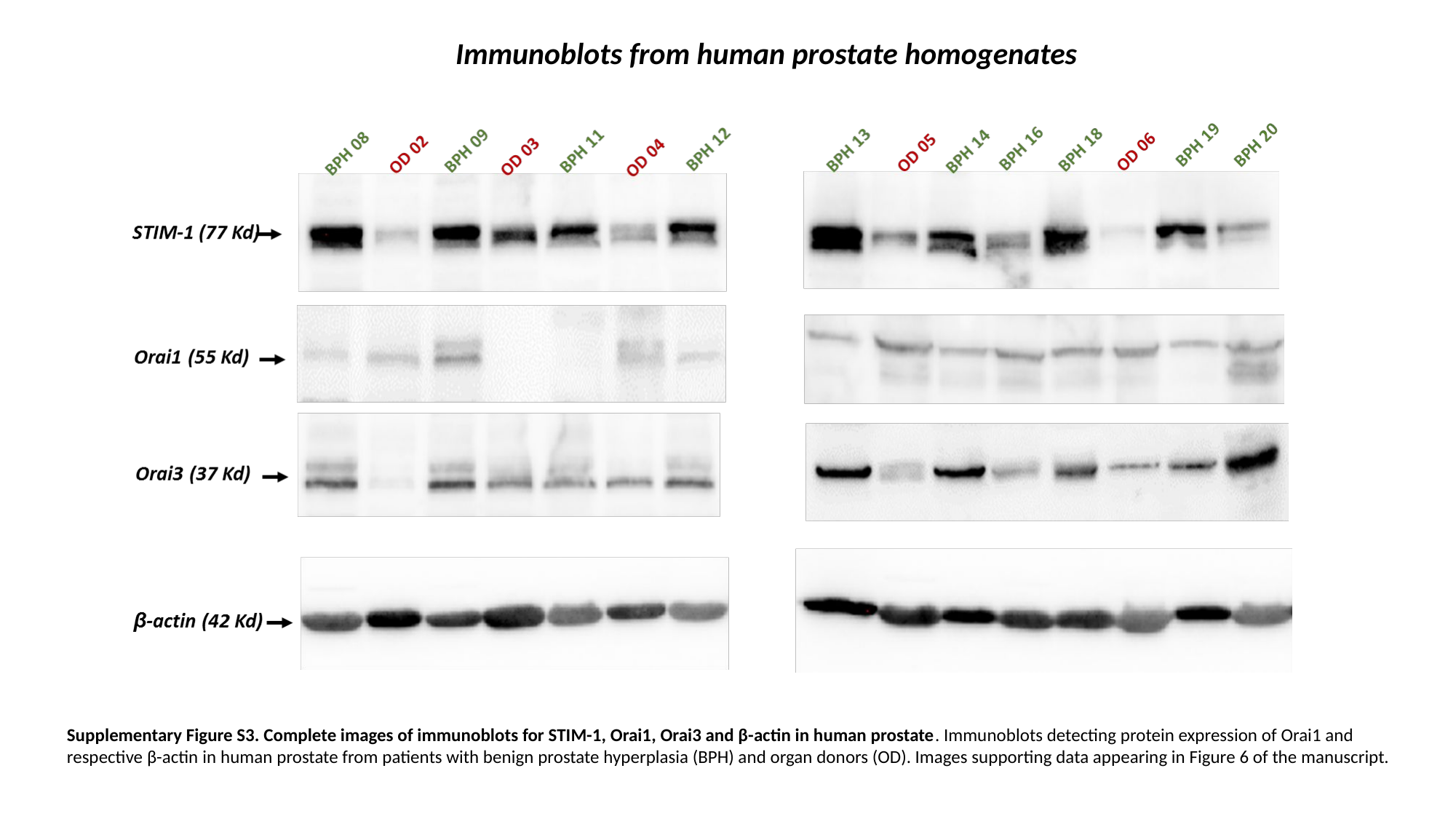

Immunoblots from human prostate homogenates
Supplementary Figure S3. Complete images of immunoblots for STIM-1, Orai1, Orai3 and β-actin in human prostate. Immunoblots detecting protein expression of Orai1 and respective β-actin in human prostate from patients with benign prostate hyperplasia (BPH) and organ donors (OD). Images supporting data appearing in Figure 6 of the manuscript.
